# Effect of diuretic infusion clinic in preventing hospitalization for patients with decompensating heart failure

**DOI:** 10.1177/2050312120940094

**Published:** 2020-07-04

**Authors:** Kamal Waheeb Alghalayini

**Affiliations:** King Abdulaziz University, Jeddah, Saudi Arabia

**Keywords:** Diuretics, infusion protocol, diagnosis, furosemide, heart failure, hospitalization, outpatient

## Abstract

**Introduction:**

It is proposed that access to administering intravenous furosemide outside the hospital can contribute to lowering hospital admissions for heart failure. This study aims to evaluate the effect of outpatient furosemide infusion protocol in preventing hospitalization for patients with decompensating heart failure. This constitutes designing a viable clinical pathway in hospitals using a multidisciplinary heart failure program.

**Methods:**

A prospective interventional study testing the effect of diuretic infusion clinic in preventing hospitalization for patients with decompensating heart failure was conducted on 150 decompensating heart failure patients requiring hospital admission. Only 105 patients met the criteria and subsequently enrolled in the study. Each patient was administered intravenous furosemide infusion one or more times according to the protocol and depending on their symptoms of decompensation. Patients were referred for admission at any point once there is no improvement of their medical condition, or referred to heart failure clinic when clinical picture improved as observed by the treating team.

**Results:**

In total, 14 of 105 patients who received intravenous furosemide infusion did not respond to diuretic infusion protocol and required hospital admission while 91 patients responded to same protocol and did not require admission, P value was statistically significant in three laboratory test measures of potassium (<0.001), urea (0.004), and creatinine (0.008). Heart failure with reduced ejection fraction was observed in 70 (76.9%) responders with a mean ejection fraction of 23% and in 9 (64.3%) non-responders with mean ejection fraction of 19.9%.

**Conclusion:**

Outpatient intravenous furosemide infusion protocol is effective in preventing hospitalization for decompensating heart failure and a viable clinical pathway for heart failure programs.

## Introduction

Heart failure (HF) is defined as a complex multifactorial clinical syndrome that leads to significant morbidity and mortality.^1^ Impaired cardiac contraction can cause fluid retention, which is an important indication of HF.^2^ In current clinical practice, lung congestion can be reduced and oxygenation can be improved through the promotion of diuresis. This is often promoted by the use of loop diuretics. Furosemide is one of the most effective loop diuretics for treating decompensated HF.^1,3^ Nevertheless, there is no general consensus regarding the mode and dosage of intravenous loop diuretics for HF patients. This variation may partly be due to a wide spectrum of HF severity, variable patient responses, and physician discretion across a variety of medical practices in different countries.^4^ Studies offering guidance about therapy and approved programs are sparse. However, despite different systems and approaches, there is general agreement that loop diuretics are an important modality of treatment for patients with decompensated HF.^1^

Intravenous injections of furosemide have a pH of 9.0 that can result in discomfort and irritation for patients. Furosemide formulation with pH of 7.4 has been commercially developed to minimize the risk of tissue irritation.^5^ The diuretics need to be administered with care and continuous monitoring. For instance, injudicious use of diuretics may cause hemodynamic instability, renal impairment, or electrolyte imbalance that may contribute toward worse prognosis and increased duration of hospitalization.^6,7^ The efficacy and safety of administering furosemide for HF patients have been investigated in a number of studies, but there is some disagreement in the results.^4,8–10^ Additional benefits of furosemide, as compared to bolus injection, are a result of the continuous infusion of furosemide because of less variability in peak plasma furosemide concentration. The decreased variability of furosemide concentration results in decreased risk of electrolyte imbalance, resulting in consistent and predictable urine output. There is a significant correlation between continuous infusion and decreased rate of mortality and shorter hospital stay as compared to administering bolus.^4^

A study conducted by Felker et al.^4^ investigated the diuretic strategies in patients with acute decompensated HF. The results showed that there was no significant difference in clinical endpoints and mortality rates between bolus injection and continuous infusion of furosemide in HF. Few previous studies have examined potentially harmful effects of continuous furosemide infusions, such as acute kidney injury, transient hypotension, and electrolyte disturbances,^4,9^ therefore, it is difficult to determine the most significant and effective methods of administering furosemide. Many HF patients experience repeated hospital admissions because of fluid overload, which is accompanied by congestive symptoms, although there has been significant improvement in the management of HF in recent years.^5^ A study conducted by Owen et al.^11^ has shown that administering furosemide intravenously is the most effective loop diuretic treatment for patients with decompensated HF.

Access to administering intravenous furosemide outside the hospital would be a potential step for developing a new care model, reducing the number of hospital admissions. This study aims to evaluate the effect of outpatient furosemide infusion protocol in preventing hospitalization for patients with decompensating HF. This would be an important step in developing a clinical pathway for hospitals applying multidisciplinary HF program.

## Methods

### Study design

A prospective interventional method was employed to identify the significance of administering outpatient furosemide infusion in preventing hospitalization for decompensating HF patients. The study was conducted from April 2018 to April 2019.

### Inclusion and exclusion criteria

No sample size calculation was applied as all patients with the following criteria in our center were selected for the study. Symptomatic HF patients presenting to HF clinic had maximal tolerated increase in oral loop diuretic consumption according to their clinical condition; patients without significant clinical improvement and requiring hospital admission are selected for this study. The inclusion criteria were as follows: decompensating patients not responding to the maximal tolerated increase in oral diuretics, signs or symptoms of heart failure including shortness of breath, orthopnea, paroxysmal nocturnal dyspnea (PND), lower limb edema or ascites, and pulmonary vascular congestion diagnosed on a chest radiograph. Patients in shock (systolic blood pressure (BP) less than 80 mm Hg), suffering from severe renal dysfunction (serum creatinine more than 4.5 mg/dL) or liver failure were excluded from this study.

### Study participants

A total of 150 patients were referred for hospital admission with decompensating HF at King Abdulaziz University Hospital, Jeddah, Saudi Arabia. Only 105 patients met the inclusion criteria.

### Ethical consideration

The study was conducted in accordance with the Declaration of Helsinki.

## Study procedure

### Clinical variables

The clinical characteristics of patients included the following: symptoms and signs of HF, risk factors as diabetes mellitus and hypertension. In addition, the laboratory investigation included tests for complete blood count (CBC), sodium, potassium, creatinine, glucose, urea, and pro-B-type natriuretic peptide (pro-BNP) concentrations on patients’ blood samples were recorded at the time of admission. Any medication taken by patients previous to admission were listed and examined.

### Clinical admission criteria

Patients complaining of shortness of breath, lower limp edema, fatigue, and gaining 3 kg in 3 days or 5 kg in 1 week; normal mental status; heart rate between 50 and 130 bpm; systolic blood pressure between 90 and 175 mm Hg; and oxygen saturation more than 90% on room air.

### Clinical admission guidelines

Nursing guidelines includes all the following steps. Verifying patient identification. Obtaining detailed history and clinical examination. Completion of nursing form. Positive vital signs and a record of height, weight, and laboratory values. Brief discussion of the procedure with the patient, allowing them to ask questions and reduce anxiety. Obtaining consent form. Insertion of peripheral intravenous (IV) line. Testing of all equipment prior to commencing the procedure. Placing the patient in a comfortable position.

### Clinical discharge guidelines

Documentation as part of the hospital policy. Vital signs should be stable and within acceptable limits for at least 1 h prior to discharge. Low salt and any other diet restrictions. Daily body weight and notify the doctor if the patient gains 3 kg in 3 days or 5 kg in a week. Indicate the appropriate activity-level based on all medical conditions. Instructions on what to do if symptoms occur, change, or worsen.

### Patients follow-up

All patients were contacted 24 h post discharge via a telephone call, standard questions for volume over load are asked with answers either improved or not (shortness of breath, night cough, orthopnea, paroxysmal nocturnal dyspnea) accordingly if patients show clinical improvement to some parameters they are asked to come again for a second session of furosemide infusion or given an appointment to the HF clinic, with maximum of 6 days if improved, for full assessment including clinical picture and furosemide side effect (tinnitus, renal function, sodium, and potassium). Patients were referred for admission at any point once there is no clinical response to furosemide infusion as observed by the treating team. A 30-day follow-up telephone call for readmission is a standard care.

Primary end point is hospital admission; secondary end points are 30 days readmission and weight loss.

### Statistical analysis

The data obtained from the patients were entered into a data sheet on Microsoft Excel. The data were then coded and entered into the Statistical Package of Social Sciences (SPSS), version 20.0. Categorical data were tabulated in the form of frequencies and percentages, and a chi-square test was applied to evaluate the level of significance.

## Results

**Figure 1.**
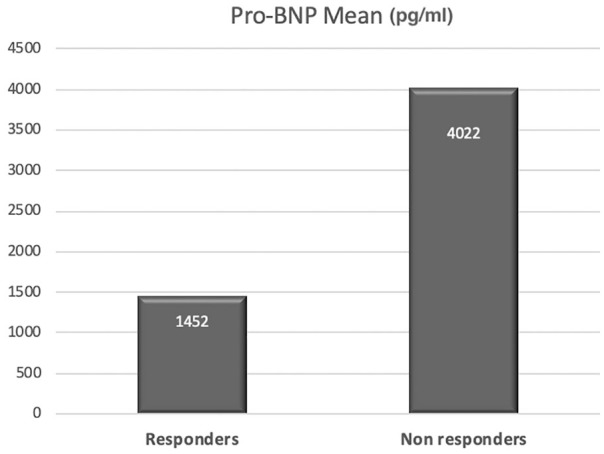
Pro-BNP mean.

Among 150 decompensating HF patients recruited for this study only 105 patients meet the inclusion criteria, 91 HF patients improved clinical condition and the intervention saved then from hospital admission (responders), and 14 patients were considered to be failure cases and required hospitalization (non-responders). The majority of the responders group (73.6%) were males, whereas 26.4% were females. About 45.1% of patients were aged between 61 and 70 years and a small number of patients (2.2%) belonged to the 81–90 years of age group. Non-responders belonged either to the 51–60 years of age group (35.7%) or the 61–70 years of age group (42.6%). Table 1 shows the two major risk factors for HF: diabetes and hypertension. Diabetes was in the majority of patients in this cohort including 83 (91%) in the responders group and 12 (85%) in the non-responders group. Hypertension showed a higher percentage than diabetes, which is well-known in the Saudi population, in the responder groups, the number of hypertensive patients are 88 (96%) while in the non-responder groups it was 13 (92%). Table 2 describes the mean baseline laboratory results of patients at the time of first infusion session. The results for white blood cells, platelets, sodium, potassium, creatinine, glucose, and urea, in the responders group were 4.8 K/µL, 195 K/µL, 134 mmol/L, 3.2 mmol/L, 117 µmol/L, 6.1 mmol/L, and 10.6 mmol/L, respectively, while in the non-responders group the results for the same variables were 6.1 K/µL, 224 K/µL, 128mmol/L, 4.4 mmol/L, 195 µmol/L, 6.8 mmol/L, and 19.6 mmol/L, respectively. P value was statistically significant in three laboratory test measures of potassium (<0.001), urea (0.004), and creatinine (0.008). Table 3 shows the correlation between number of infusion sessions and weight loss, responders were 91 patients, 48 (52.75%) patients received one infusion session and had a mean weight loss of 2.58 kg, while 37 (40.66%) patients received two infusion sessions with a mean weight loss of 3.39 kg, finally for the responders a total of six (6.59%) patients received three infusion sessions with a mean weight loss of 3.91 kg. All 14 non-responders are in two groups, first group includes eight (57.14%) patients receiving one infusion session with a mean weight loss of 1.44 kg, while the second group includes six (42.86%) patients receiving two sessions of furosemide infusion with a mean weight loss of 2.13 kg. Table 4 shows the types of HF among the patients population according to European Society of cardiology. HF with reduced ejection fraction (HFrEF) was in 70 (76.9%) in responders with a mean ejection fraction of 23% and in 9 (64.3%) non-responders with mean ejection fraction of 19.9%. HF with midrange ejection fraction (HFmrEF) was in 13 (14.3%) responders with a mean ejection fraction of 42.1% and in 3 (21.4%) non-responders with mean ejection fraction of 41.9%. HF with preserved ejection fraction (HFpEF) was in 8 (8.8%) responders with a mean ejection fraction of 55.4% and in 2 (14.3%) non-responders with mean ejection fraction of 60.3%. N-terminal (NT)-Pro BNP was collected for all 105 patients, the responders’ group mean results were 1452 pg/mL and the non-responders’ group mean results were 4022 pg/mL (Figure 1).

**Table 1.** Diabetes and hypertension among patients cohort.

Item	Responders *N* (%)	Non-responders *N* (%)
**Diabetes**	83 (91.2%)	12 (85.7%)
**Hypertension**	88 (96.7%)	13 (92.9%)

**Table 2.** Laboratory results at the time of first infusion session.

		Responders	Non-responders	
Item	Measure	Mean	Mean	P value
**Characteristics**	WBCs (K/µL)	4.8	6.1	0.191
	Platelets (K/µL)	195	224	0.432
	Na^+^ (mmol/L)	134	128	0.329
	K^+^ (mmol/L)	3.2	4.4	<0.001
	Creatinine (µmol/L)	117	195	0.008
	Glucose (mmol/L)	6.1	6.8	0.571
	Urea (mmol/L)	10.6	19.1	0.004

WBCs: white blood cells.

**Table 3.** Correlation between number of infusion sessions^a^ and weight loss.

	No. of infusion sessions^a^	No. of patients	Mean weight loss (kg)
**Responders (91)**	1	48 (52.75%)	2.58
	2	37 (40.66%)	3.39
	3	6 (6.59%)	3.91
**Non-responders (14)**	1	8 (57.14%)	1.44
	2	6 (42.86%)	2.13

a Infusion session is 5–6 h per session.

**Table 4.** Types of HF among responders and non-responders.

Hear failure type	Responders		Non-responders	
	*N* (%)	Ejection fraction mean	*N* (%)	Ejection fraction mean
HFrEF	70 (76.9%)	23.3%	9 (64.3%)	19.9%
HFmrEF	13 (14.3%)	42.1%	3 (21.4%)	41.9%
HFpEF	8 (8.8%)	55.4%	2 (14.3%)	60.3%

HFrEF: heart failure with reduced ejection fraction, HFmrEF: heart failure with midrange ejection fraction, HFpEF: heart failure with preserved ejection fraction.

Table 1 shows the two major risk factors for HF: diabetes and hypertension. Diabetes was in the majority of patients in this cohort: 83 (91%) in the responders group and 12 (85%) in the non-responders group. Hypertension showed a higher percentage than diabetes, which is well-known in the Saudi population, in the responders group the number of hypertensive patients were 88 (96%) while in the non-responders group it was 13 (92%).

Table 2 describes the mean baseline laboratory results of patients at the time of first infusion session. The white blood cells, platelets, sodium, potassium, creatinine glucose, and urea in the responders group were 4.8 K/µL, 195 K/µL, 134 mmol/L, 3.2 mmol/L, 117 µmol/L, 6.1 mmol/L, and 10.6 mmol/L, respectively, while in the non-responders group the results for the same variables were 6.1 K/µL, 224 K/µL, 128 mmol/L, 4.4 mmol/L, 195 µmol/L, 6.8 mmol/L, and 19.6 mmol/L, respectively.

Table 3 shows the correlation between number of infusion sessions and weight loss, responders were 91 patients, 48 (52.75%) patients received one infusion session and had a mean weight loss of 2.58 kg, while 37 (40.66%) patients received two infusion sessions with a mean weight loss of 3.39 kg, finally for the responders a total of six (6.59%) patients received three infusion sessions with a mean weight loss of 3.91 kg. All 14 non-responders are in two groups, first group 8 (57.14%) patients received one infusion session with a mean weight loss of 1.44 kg, the second group of six (42.86%) patients received two sessions of furosemide infusion with a mean weight loss of 2.13 kg.

Table 4 shows the types of HF among the patients population according to European Society of Cardiology. HFrEF was in 70 (76.9%) responders with a mean ejection fraction of 23% and in 9 (64.3%) non-responders with mean ejection fraction of 19.9%. HFmrEF was in 13 (14.3%) responders with a mean ejection fraction of 42.1% and in 3 (21.4%) non-responders with mean ejection fraction of 41.9%. HFpEF was in 8 (8.8%) responders with a mean ejection fraction of 55.4% and in 2 (14.3%) non-responders with mean ejection fraction of 60.3%.

A limitation in this study includes single-center patient’s collection and relatively small but representable number of patients.

## Discussion

The study has investigated the effectiveness of diuretic infusion clinic in preventing hospitalization for patients with decompensating HF. The results shows a significant reduction in hospital admissions in the sample examined. A study conducted by Sica et al.^5^ suggests that the administering conventional furosemide through intravenous injection at a slow infusion rate does not cause any discomfort in the patient. HF patients who did not show a significant response to oral diuretics were given parenteral diuretics to treat the disease effectively. HF patients required escalation of oral diuretics along with its dosing frequency because it is characterized by unpredictable periods of decompensation. Therefore, intravenous diuretics were prescribed for the cases where adjustment in oral treatment failed.^5^ In Sica et al.,^5^ the impaired absorption results reduced response to oral medication for a short period of time as a result of fluid overload or impairment in the absorptive function of the stomach and intestine. There was increased variability in the average bioavailability of furosemide after its oral administration, with a range of 49%–72%. In the majority of patients, parenteral furosemide therapy reduces hypervolemia and helps in the restoration of oral bioavailability back to oral maintenance therapy. Sica et al.^5^ showed that peak levels of furosemide are generally achieved within 30–60 min of administration as the therapeutic plasma levels are achieved. Sica et al. showed that it is important to maintain the plasma levels of furosemide in therapeutic range until the next intravenous administration. Therefore, both Sica et al. and this study have found that patients can be discharged earlier or prevent admission when they can receive parenteral diuretic administration in an outpatient setting. In our study, each patient was administered intravenous furosemide infusion, starting with 20 mg/h and increase 10 mg/h every hour guided by a systolic blood pressure more than 95 mm Hg, average infusion time was 6 ± 1 h. Infusion was discontinued if patient can no more tolerate the medication (intolerance is defined as systolic blood pressure of less than 95 mm Hg and dizziness despite decreasing the dose of medication infusion). This was repeated on alternative days until the symptoms improve or patient is referred for admission.

Burdens on the healthcare system and patients’ family can be reduced as the clinicians test specific workflows associated with administering furosemide. This is likely to result in increased prevalence of home-based treatment as an alternative to inpatient care. Home-based treatment is marked with shortened length of stay at the hospital. This study has investigated the effectiveness of daycare furosemide infusion protocol as an add-on strategy for gaining support and providing information to minimize the need for hospitalization.

Furosemide works by inhibiting the sodium–potassium–chloride co-transporter in the apical membrane of tubular epithelial cells.^12^ These epithelial cells are present in the thick ascending limb of the loop of Henle that is responsible for absorbing significant amounts of sodium in the glomerular filtrate. It is this absorption that results in the inhibition of the sodium–potassium–chloride co-transporter causing diuresis and potent natriuresis. A significant increase in the diuretic response is achieved by maintaining a constant amount of diuretic at the site of action through continuous intravenous infusion of furosemide.^13^ This administration also limits the compensatory retention of sodium that can occur with subsequent doses of furosemide.^13^ Decisions about timing of doses can be simplified and the chance of medicine administration errors mitigated through continuous infusion of furosemide. It is important to monitor the adverse effects associated with administration because fluctuations in intravascular volume may cause electrolyte abnormalities, enhance tolerance, interfere with hemodynamic stability, increase toxicity, and cause renal failure.^14^

This study has demonstrated that increased weight loss by administering furosemide infusion is associated with improved outcomes, consistent with a previous study,^15^ and a study conducted by Ng and Yap,^16^ which showed a significant association between continuous infusion of furosemide and increased loss of body weight. It has been shown that increasing the dose of diuretic helps to maintain the therapeutic effect which is a major risk to diuretic resistance. However, the urine output was not assessed in this study. To avoid discomfort and risk of infection, catheterization was not performed on patients for measurement and recording of urine output; body weight is a better indication of diuretic effect compared to total urine output. However, a similar study conducted by Shah et al.^17^ showed significant association between continuous furosemide infusion and the increase in total urine output. This study has helped in determining a viable alternative clinical pathway, showing the effectiveness of the outpatient diuretic room in reducing emergency department admission—often including 30 days readmission thereafter.

The prognosis of HF patients is favorable considering the high number of admissions. Therefore, almost all HF programs are developed considering various strategies to protect patients from deteriorating, while aiming to lower hospital admissions and emergency room visits. Furosemide is a key therapy in controlling HF symptoms when the condition of patients deteriorates. Proper absorption of oral medication can be prevented as the result of gastrointestinal tract edema, which often makes intravenous administration the ideal route for delivery.

Limitation of this study is the absence of a control group due to the assumption of hospital admission for all patients, absence of sample size calculation, and single-center patient collection.

## Conclusion

This study has evaluated the effect of diuretic infusion clinic in preventing hospitalization for patients with decompensating HF. The study has shown justification for designing an alternative clinical pathway for decompensating HF patients, minimizing hospital admissions and maintaining ambulatory status through the addition of an outpatient diuretic room, leading to improved quality of life and potentially a reduction in healthcare costs and mortality.
